# Potential applications of JAK inhibitors, clinically approved drugs against autoimmune diseases, in cancer therapy

**DOI:** 10.3389/fphar.2023.1326281

**Published:** 2024-01-03

**Authors:** Xiao-Huan Wei, Yuan-Yuan Liu

**Affiliations:** ^1^ Respiratory and Critical Care Department, Xuzhou Central Hospital, Xuzhou, Jiangsu, China; ^2^ Xuzhou Clinical School of Xuzhou Medical University, Xuzhou, Jiangsu, China; ^3^ Oncology Department, People’s Hospital of Peixian, Xuzhou, Jiangsu, China

**Keywords:** autoimmune diseases, JAK inhibitors, clinical translation, anti-cancer, immunotherapeutic therapy

## Abstract

Disturbances in immunoregulation may lead to both cancer and autoimmune diseases. Many therapeutic drugs for autoimmune diseases also display anti-tumor efficacy. The Janus kinase/signal transducer and activator of transcription signaling pathways are involved in the secretion of more than 50 distinct cytokines, which have critical roles in inducing autoimmune diseases and tumorigenesis. Thus, Janus kinases have become classical immunotherapeutic targets for immune disease. More than 70 Janus kinase inhibitors have been approved as immunomodulatory drugs for clinical use, of which 12 are used in the treatment of autoimmune diseases. This systematic review aims to elucidate the anti-tumor role of clinically approved Janus kinase inhibitors that were primarily designed for the treatment of autoimmune diseases and their potential for clinical translation as cancer treatments.

## 1 Introduction

Chronic inflammation is often associated with autoimmune diseases (AIDs) and cancer. Only 5%–10% of cancers are caused by an inherited gene defect, with the remaining 90%–95% resulting from environment- or lifestyle-induced chronic inflammation (Hirano, 2021). In general, immune cells target and kill cancer cells in the early stage of tumorigenesis. However, during crosstalk with the tumor microenvironment, they are “domesticated,” thereby losing their ability to eliminate cancer cells and possibly even facilitating the progression of tumors (Lei et al., 2020). Harnessing immune cells has been widely explored as a powerful strategy to inhibit cancer in both clinical and pre-clinical studies (Yang, 2015). Various drugs targeting immune cells that are used in the treatment of immunological diseases have also been explored with respect to their potential role in anti-cancer therapy. In this review, we summarize the effects on tumorigenesis and potential use in cancer therapy of clinically approved JAK inhibitors that were primarily designed for treatment of AIDs.

The immune system plays a critical part in the maintenance of individuals’ health. Immune deficiency may lead to an inability to activate the necessary response to protect against pathogen invasion, whereas immune overactivation may cause AIDs. There are nearly 100 distinct AIDs, affecting approximately 3% of the population (Youinou et al., 2010). In general, these can be divided into two main categories: organ-specific AIDs, which affect organs including the inner ear, skin, thyroid and parathyroid gland, heart, liver, adrenal gland, pancreas, gastrointestinal system, reproductive system, and connective tissue; and systemic AIDs, which may affect the cardiovascular, hematopoietic, and neurological systems (Wang et al., 2015).

The synthetic drugs approved to date for the treatment of AIDs are dominated by JAK inhibitors (Schwartz et al., 2017). This is because the Janus kinase/signal transducer and activator of transcription (JAK/STAT) signaling pathway is involved in the mediation of more than 50 distinct cytokines, and elevated cytokine expression caused by excessive activation of the JAK/STAT signaling pathway is a decisive factor in the occurrence of AIDs (O'Shea et al., 2002). Cytokines are also critical for malignant cell growth (Kontzias et al., 2012); thus, almost of those drugs have also been investigated with respect to their ability to treat cancer.

## 2 Relationship between AIDs and cancer

**FIGURE 1:**
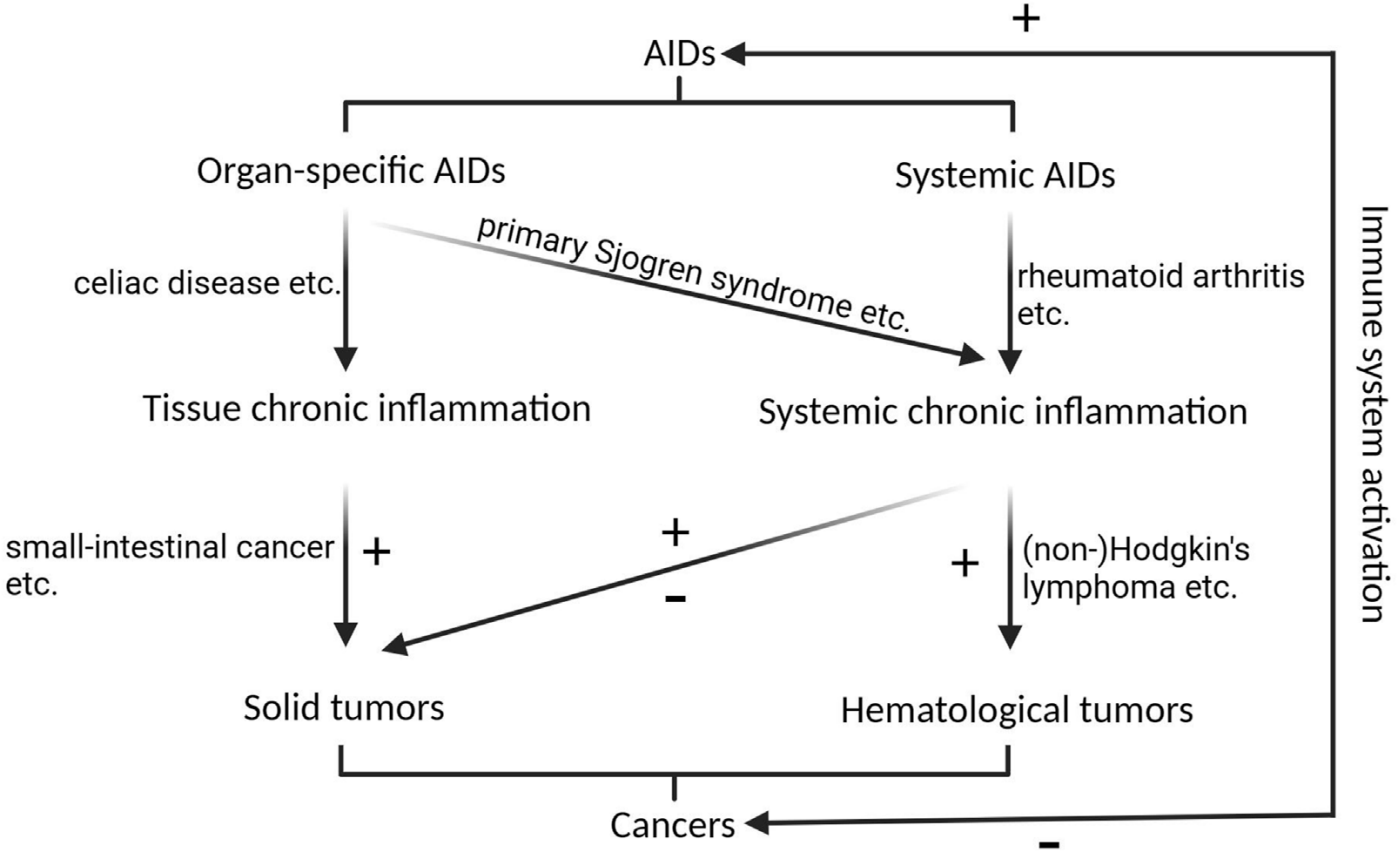
The correlation between AIDs and cancers. Organ-specific AIDs primarily contribute to solid tumorigenesis by inducing chronic inflammation in the affected tissues, while skin-specific AIDs, like primary Sjogren syndrome, can trigger systemic chronic inflammation, elevating the susceptibility to both overall solid tumors and lymphomas. Systemic AIDs increase the risk of hematological malignancies, and their impact on solid malignancies is multifaceted. Activation of immune system inhibits cancers, while promotes AIDs. + indicates promotional role on tumorigenesis or AIDs, - indicates inhibitory role on tumorigenesis.

The association between AIDs and cancer is well established and has been systematically summarized in previous reviews (Giat et al., 2017), and we briefly summarized the association between AIDs and cancer as indicated in Figure 1. In brief, organ-specific AIDs tend to induce tissue tumors, possibly owing to chronic inflammation around the tissues. For example, celiac disease is a gastrointestinal system AID that leads to difficulty in digesting food and carries a high risk of small-intestinal cancer (Green et al., 2003); and a skin-specific AIDs, primary Sjogren syndrome, increases the risk of overall solid tumors and lymphomas, especially non-Hodgkin’s lymphoma (NHL), for which the risk may well exceed a ten-fold increase (Smedby et al., 2006), possibly owing to systemic chronic inflammation.

Systemic AIDs tend to affect both hematological malignancies and organ-specific solid tumors. For example, systemic lupus erythematosus increases the risk of cancer in various organs, while decreasing the risk of others (Mao et al., 2016). Patients with rheumatoid arthritis (RA) have an increased risk of hematological malignancies, including Hodgkin’s lymphoma and NHL; however, they are at decreased risk of most solid malignancies, including kidney, liver, prostate, gynecological, and gastric cancers (Giat et al., 2017).

The associations of other AIDs, such as polymyalgia rheumatica and giant cell arteritis, with cancer are not clear. Some studies have reported an increased risk of cancer in patients with polymyalgia rheumatica and giant cell arteritis (Ji et al., 2010; Muller et al., 2014), whereas others report no association or even a reduced risk (Kermani et al., 2010a; Kermani et al., 2010b).

Studies have also illustrated the phenomenon of autoimmunity secondary to malignancy and the co-occurrence of cancer and AIDs. Treatment of cancer patients by activating an immune reaction could lead to AIDs, especially in patients with pre-existing AIDs. There have been reports that more than 30% of such patients experience AIDs relapses or develop new autoimmune manifestations (Coureau et al., 2020).

## 3 Potential anti-tumor role of JAK inhibitor drugs approved for AIDs treatment

Cancer and AIDs are closely related, as discussed above; thus, many drugs that have been clinically approved for treatment of AIDs have also been investigated with respect to their potential roles in the treatment of cancer. JAK/STAT signaling pathways are the classical immunotherapeutic targets. There are four JAKs, JAK1-3 and TYK2 (tyrosine kinase 2), and seven STATs, STAT1/2/3/4/6 and STAT5A/5B, in humans (Roskoski, 2023a). The regulatory role of the JAK/STAT signaling pathway in AIDs has been extensively summarized in a previous review (Xue et al., 2023). Thus, inhibition the of JAK/STAT pathway is widely used to treat AIDs.

**FIGURE 2:**
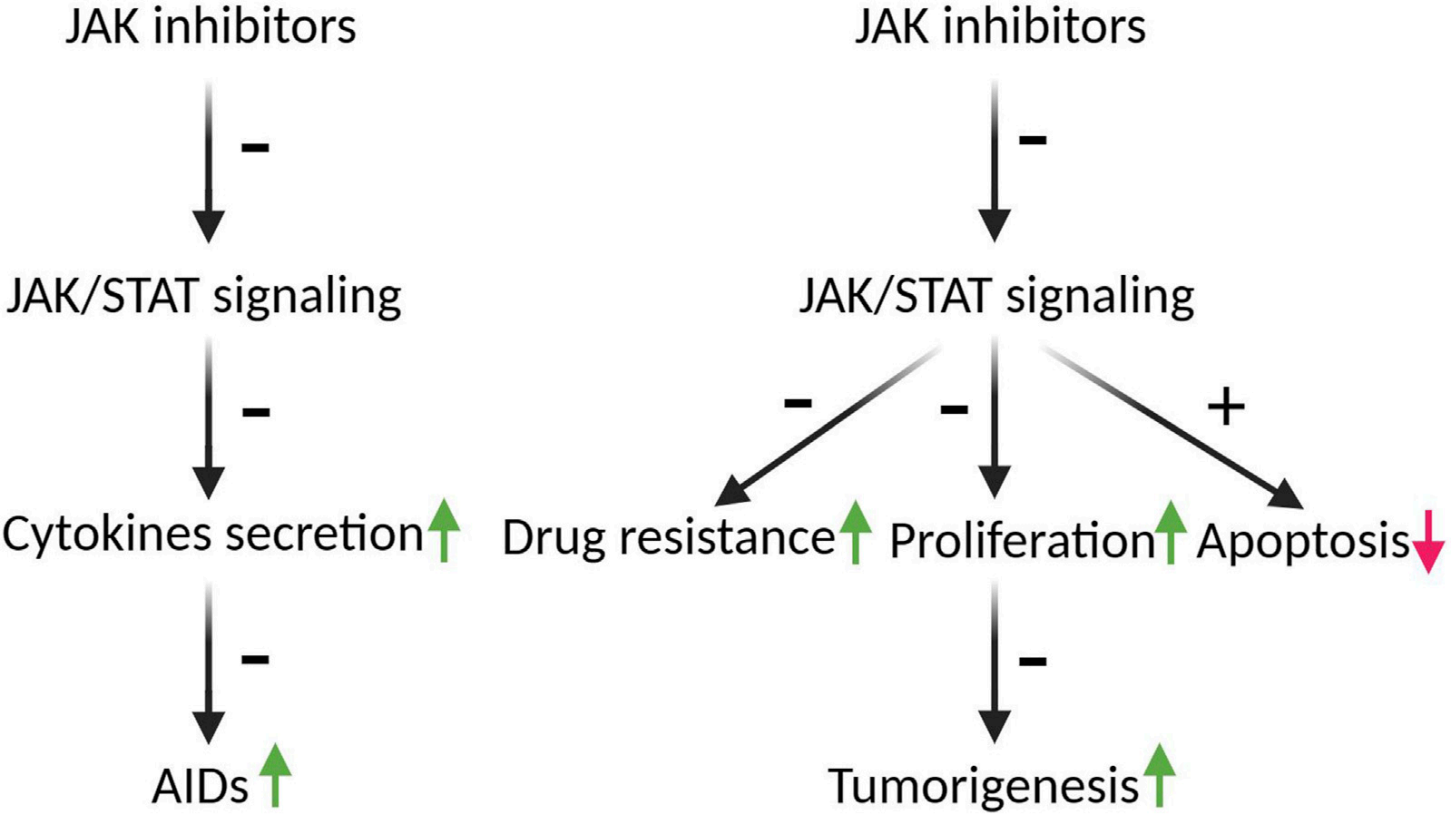
The schematic depicting the mechanism of action of JAK inhibitors in the progression of AIDs and cancers. The JAK/STAT pathways play a crucial role in the secretion of more than 50 distinct cytokines, which play pivotal roles in the development of AIDS and tumorigenesis. Inhibition of JAK/STAT pathways by JAK inhibitors mitigates AIDs by decreasing cytokines secretion. Moreover, hyperactivation of JAK/STAT is implicated in drug resistance and the survival of tumor cells. JAK inhibitors have the potential to counteract tumorigenesis by reversing drug resistance, inducing G2 arrest, and augmenting apoptosis. Arrows indicate the roles of JAK/STAT in AIDs and tumorigenesis, green arrows indicate promotion, red arrow indicates inhibition; ± indicate the roles of JAK inhibitors in AIDs and tumorigenesis, +means promotion, - means inhibition.

The first generation of JAK inhibitors, known as jakinibs, bind to the kinase domain of JAKs. To date, 72 small-molecule protein kinase inhibitors have been approved by the US Food and Drug Administration (FDA) (Roskoski, 2023b), of which 12 JAK inhibitors have been approved for clinical use against AIDs; these comprise ruxolitinib, pacritinib, fedratinib, tofacitinib, baricitinib, abrocitinib, filgotinib, oclacitinib, peficitinib, upadacitinib, deucravacitinib, and delgocitinib (Shawky et al., 2022; Roskoski, 2023a; Li et al., 2023). Various mutations of JAKs or overactivation of JAK/STAT signaling pathways also been reported in various malignant tumors, including hematological and solid tumors; thus, most of the jakinibs also been demonstrated have anti-tumor efficacy, and scheme with JAK inhibitors mechanism of action in the course of AIDs and cancers was shown in Figure 2.

### 3.1 Ruxolitinib

Ruxolitinib, formerly known as INCB018424 or INC424, a selective oral inhibitor of JAK1 and JAK2 (Ajayi et al., 2018), was approved for treatment of myelofibrosis (MF) by the FDA in 2011 and by the European Medicines Agency (EMA) in 2012 (Mascarenhas and Hoffman, 2012) for polycythemia vera (PV) (2014) (Raedler, 2015) and acute and chronic graft *versus* host disease (GVHD) (2021) (Yang et al., 2021). Clinical research has demonstrated that it benefits some patients with pancreatic cancer (American Association for Cancer Research, 2015). Ruxolitinib has also resulted in positive response in patients with head and neck cancer with STAT3 overactivation (Qureshy et al., 2022). Systematic screening of T cell acute lymphoblastic leukemia (T-ALL) genomes revealed activating mutations in JAK1, JAK3, and STAT5 in 20%–30% of T-ALL cases (Belver and Ferrando, 2016), and ruxolitinib has displayed significant anti-tumor efficacy against T-ALL in both primary xenograft models and clinical trials (Lato et al., 2021; Moskowitz et al., 2021; Kolodrubiec et al., 2022). Patients with chronic neutrophilic leukemia (CNL) and atypical chronic myeloid leukemia (aCML) have also shown positive responses to ruxolitinib (Dao et al., 2020). In T-cell prolymphocytic leukemia, ruxolitinib promotes apoptosis and shows synergistical efficacy in combination with venetoclax, a B-cell lymphoma-2 inhibitor (Herbaux et al., 2021). Ruxolitinib combined with other mitogen-activated protein kinase/extracellular signal-regulated kinase inhibitors have been shown to overcome therapeutic resistance and promote immune checkpoint therapy in pancreatic ductal adenocarcinoma (PDAC) (Datta et al., 2022). Ruxolitinib and calcitriol combination treatment showed synergistic anti-cancer effects on some breast cancer cell lines (Schneider et al., 2022), and combinations of ruxolitinib with SMO-GLI1/tGLI1 pathway inhibitors synergistically inhibited growth of triple-negative breast cancer and human epidermal growth factor receptor-2 (HER2)-positive breast cancer both *in vitro* and *in vivo* (Doheny et al., 2020). Other studies have also reported anti-solid-tumor effects of ruxolitinib alone or in combination against various cancers, including metastatic lung cancer (Taverna et al., 2020), non-small-cell lung cancer (Patel et al., 2019), hepatocellular carcinoma (Wilson et al., 2013), and myeloproliferative neoplasms (MPN) (Eliacik et al., 2015; Wang et al., 2023).

### 3.2 Pacritinib

Pacritinib (SB1518), an inhibitor against JAK2 and mutationally activated JAK2 (JAK2V617F), was approved for treatment of MF by the FDA in 2022 (Shawky et al., 2022). Oral administration of pacritinib in murine models of acute myeloid leukemia (AML) led to significant inhibition of primary tumor growth and lung metastasis (Hart et al., 2011). Pacritinib also decreased the viability of patient-derived initiating cells of glioblastoma multiforme (GBM) *in vitro* at low micromolar doses, as well as improving response to tumozolomide in tumozolomide-resistant glioblastoma multiforme and thus improving overall median survival of mice in an orthotopical xenograft model (Jensen et al., 2017). Furthermore, pacritinib suppresses the expression of checkpoint proteins; a preclinical trial and a pilot phase I study of pacritinib and chemotherapy in FLT3-ITD-positive AML found that combination therapy was well tolerated, and preliminary results indicated anti-leukemic activity in patients with FLT3 mutations (Jeon et al., 2020). Clinical trials have also been performed to explore the benefits of pacritinib treatment for patients with metastatic refractory colorectal adenocarcinoma (CRC); however, no objective response was achieved (Chen et al., 1997). Elevated glucose consumption has a critical role in maintaining the growth of squamous cell lung cancer; pacritinib reduced glucose consumption in squamous cell lung cancer by inhibiting hexokinase activity (Ghezzi et al., 2023). Synergistic effects of pacritinib with other treatments have also been widely investigated. A combination of pacritinib with histone deacetylase (HDAC) inhibitor pracinostat (SB939) led to synergistic effects on tumor growth and a reduction of metastasis in AML (Novotny-Diermayr et al., 2012). In PC3/ER3 xenografts, a combination of pacritinib with erlotinib (ELTN), an epidermal growth factor receptor tyrosine kinase inhibitor, showed synergistic effects on tumor shrinkage by suppressing MET (Ochi et al., 2016). Combined treatment with pacritinib and SMO inhibitors (vismodegib and sonidegib) synergistically inhibited growth of triple-negative breast cancer and HER2-positve trastuzumab-resistant BT474-TtzmR cells both *in vitro* and *in vivo*. The combination therapy also synergistically inhibited breast cancer stem cells and suppressed lung metastasis in an orthotopic BT474-TtzmR xenograft model (Doheny et al., 2020). Overexpression of P-glycoprotein (P-gp) in cancer cells leads to multidrug resistance. Co-treatment with low-dose pacritinib induced G2 arrest, reduced cell viability, and greatly increased apoptosis of P-gp overexpressing cancer cells with multidrug resistance, indicating sensitization of P-gp-overexpressing drug-resistant cancer cells (Oh et al., 2022a). Pacritinib treatment overcame resistance to paclitaxel in nasopharyngeal carcinoma (NPC) by blocking IRAK1; and combination treatment with pacritinib and paclitaxel exhibited a superior anti-tumor effect (Liu et al., 2021).

### 3.3 Fedratinib

Fedratinib (INREBIC^®^), an oral selective kinase inhibitor against both wild-type JAK2 and JAK2V617F, was approved for the treatment of adult patients with intermediate-1 or high-risk primary or secondary MF in 2019 by the FDA (Blair, 2019). Kirsten rat sarcoma 2 viral oncogene homolog (KRAS) is the major driver mutation gene for PDAC tumorigenesis. Liu et al. predicted that fedratinib would exhibit KRAS-dependent anti-cancer activity in PDAC cells based on mining of bioinformatics data (Liu et al., 2020). Activation of the JAK2/STAT3 signaling pathway induces acquired ELTN resistance; fedratinib reversed this ELTN resistance by downregulation of JAK2/STAT3 signaling, thereby ameliorating the anti-cancer effects of ELTN in non-small cell lung cancer (NSCLC) (Chen et al., 2018). Furthermore, the JAK/STAT pathway leads to HDAC inhibitor resistance in cancers; novel JAK inhibitors based on a fedratinib moiety also suppressed tumor growth of acute erythroid leukemia (AEL) and NSCLC with HDAC inhibitor resistance (Qiu et al., 2023). Fedratinib further sensitizes P-gp-overexpression-induced drug resistance, and co-treatment with anti-mitotic drugs has been shown to increase the cytotoxicity of fedratinib to KBV20C oral cancer cells by reducing cell viability, increasing G2 arrest, and upregulating apoptosis (Oh et al., 2022b). In brief, the functions of fedratinib, including the inhibition of cell activity and drug resistance in cancer therapy, have been investigated to a certain extent.

### 3.4 Tofacitinib

Tofacitinib, an oral selective inhibitor of JAK1, JAK3, and (to a certain extent) JAK2 has been approved for treatment of RA (2012) (Coricello et al., 2020), psoriasis arthritis (PsA) (2017) (Ayala-Aguilera et al., 2022), ulcerative colitis (UC) (2018) (Ayala-Aguilera et al., 2022), juvenile idiopathic arthritis (JIA) (2020) (Kostik et al., 2022), and ankylosing spondylitis (AS) (2021) (Mohanakrishnan et al., 2022). Preclinical studies have reported that tofacitinib is effective in T-ALL patients with JAK1/JAK3 mutations (Girardi et al., 2017). However, AIDs are treated by decreasing the immune response, whereas tumorigenesis may occur during immune suppression conditions; thus drugs that inhibit immune response may also lead to tumorigenesis. There have been reports that therapy with tofacitinib increases the risk of tumorigenesis compared with tumor necrosis factor (TNF) inhibitor therapy (Ytterberg et al., 2022; Curtis et al., 2023). However, a meta-analysis of observational studies found no increased risk of malignancy in patients with RA treated with tofacitinib therapy compared with those receiving conventional synthetic disease-modifying anti-rheumatic drugs or TNF inhibitor therapy (Benucci et al., 2022). Thus, the association of tofacitinib with tumorigenesis is controversial.

### 3.5 Baricitinib

Baricitinib, an oral selective JAK1/2 inhibitor, was approved as a monotherapy for the treatment of RA in 2017 by the EMA and in 2018 by the FDA (Markham, 2017; Coricello et al., 2020), as well as in combination with methotrexate (Taylor et al., 2023). Baricitinib was also approved as a treatment for COVID-19 in 2020 by the EUA and in 2022 by the FDA (Shawky et al., 2022). The efficacy of baricitinib as a cancer therapy has rarely been considered so far, although one has study reported that baricitinib did not induce apoptosis of T-ALL (Akahane et al., 2017), whereas synergistic effects for its combination with docetaxel were observed in androgen-receptor-negative prostate cancer cells (Nalairndran et al., 2021). Potential effects on the risk of tumorigenesis have also been evaluated; one study reported that the malignancy rate in RA patients treated with baricitinib was high but not significantly different from that of the general population or of patients treated with TNF inhibitors (Uchida et al., 2023). Another study has evaluated the long-term safety of baricitinib in RA patients; at present, the data do not show an increased risk of malignancy (Taylor et al., 2022).

### 3.6 Abrocitinib

Abrocitinib, an inhibitor of JAK1 and JAK2, was approved for the treatment of adult patients with refractory and moderate-to-severe atopic dermatitis (AD) in 2022 by the FDA; it was also approved by the European Commission in 2021 (Deeks and Duggan, 2021; De, 2023). The anti-tumor efficacy of abrocitinib has not yet been investigated. Its safety has been evaluated by a long-term observation study, during which three patients (0.3%) treated with 100 mg abrocitinib and four patients (0.2%) treated with 200 mg abrocitinib developed nonmelanoma skin cancer, and there were three events of adjudicated malignancies, including two of prostate cancer and one of gastric adenocarcinoma, indicating a slight increase in cancer risk among patients receiving abrocitinib treatment (Simpson et al., 2021).

### 3.7 Filgotinib

Filgotinib, a specific inhibitor targeting JAK1, was approved for the treatment of RA by the EMA in 2020 (Dhillon and Keam, 2020). JAK1/STAT3 activation leads to targeted drug resistance of NSCLC, and inhibition of the JAK1/STAT3 signaling pathway by filgotinib reversed resistance to targeted drugs (Shien et al., 2017). No malignancies (solid tumor or lymphoma) were observed in clinical trials of filgotinib treatment (Biggioggero et al., 2019). During a long-term safety clinical trial for up to 4 years, only one case of NHL was considered to be related to filgotinib treatment among 739 enrolled patients (Kavanaugh et al., 2021).

### 3.8 Oclacitinib

Oclacitinib (Apoquel^®^), a specific inhibitor targeting JAK1, was approved for the treatment of canine allergic dermatitis (CAD) in 2013 (Gonzales et al., 2014). To achieve synergistic anti-cancer activity, combination therapies of oclacitinib with cytotoxic chemotherapy, including carboplatin and doxorubicin, have been investigated in multi-pulmonary metastasis models; results indicate that oclacitinib is well tolerated in combination with carboplatin or doxorubicin, although whether anti-tumor efficacy is enhanced has not been determined (Barrett et al., 2019). Long-term treatment with oclacitinib did not appear to increase the risk of malignancy in dogs (Cosgrove et al., 2015; Lancellotti et al., 2020).

### 3.9 Peficitinib

Peficitinib, a pan-JAK inhibitor, was approved for treatment of RA in Japan in 2019 (Markham and Keam, 2019). Peficitinib has been proved to have an inhibitory effect on cancer cells that is dependent on JAK/STAT signaling. Chromatin assembly factor 1 subunit A was shown to promote the proliferation and growth of epithelial ovarian cancer cells by activating the JAK2/STAT3 signaling pathway, and peficitinib decreased cancer growth by inhibition of this pathway (Xia et al., 2023). Treatment with peficitinib inhibited octamer-binding transcription factor 4-induced promotion of viability, invasion, and tumorigenesis of ovarian cancer side-population cells (Ruan et al., 2019).

### 3.10 Upadacitinib

Upadacitinib is an oral JAK1 inhibitor that has been approved for treatment of RA (2019) (Duggan and Keam, 2019), PsA (2021) (Muensterman et al., 2022), AD (2022) (Shawky et al., 2022), and UC (2022) (Shawky et al., 2022). Induction of inflammation is a factor leading to cisplatin-induced renal and hepatic dysfunction. It has been reported that upadacitinib protects against renal and hepatic dysfunction induced by cisplatin without impairing its efficacy against breast cancer and NSCLC. Moreover, upadacitinib promoted the potency of cisplatin against lung cancer cells (Anbar et al., 2022). No malignancies (hematoma or solid tumor) were observed in clinical trials to assess the safety of filgotinib therapy, whereas seven cases (*versus* three in the placebo group) were described in patients treated with upadacitinib, indicating that upadacitinib may increase the risk of tumorigenesis (Genovese et al., 2018; Biggioggero et al., 2019).

### 3.11 Deucravacitinib

Deucravacitinib (Sotyktu™), a specific inhibitor against TYK2, was approved for treatment of moderate-to-severe plaque psoriasis (PP) in 2022 by the FDA (Papp et al., 2018). Overexpression of TYK2 occurs in the majority of malignant peripheral nerve sheath tumors (MPNST); inhibition of TYK2 by deucravacitinib decreased proliferation and induced apoptosis of these tumors through decreased expression of proteins involved in the cell cycle, mitotic, and glycolysis pathways (Borcherding et al., 2023). It has been reported that therapeutic TYK2 inhibition may increase the risk of lung cancer and NHL; however, the safety profile of deucravacitinib has not yet been determined (Yarmolinsky et al., 2022).

### 3.12 Delgocitinib

Delgocitinib (Corectim^®^), a nonselective inhibitor that inhibits all members of the JAK family, including JAK1, JAK2, JAK3, and TYK2, was approved for treatment of AD in 2020 in Japan (Dhillon, 2020). However, the anti-tumor efficacy and the risk of tumorigenesis in AIDs patient treated with delgocitinib have not yet been established.

## 4 Summary

Both cancer and AIDs are related to immune diseases (Yasunaga, 2020). Based on these associations, drugs that were designed for the treatment of AIDs have also been extensively explored with respect to their potential to treat cancers. The summary of approved JAK inhibitors for AIDs and their exploration in understanding anti-cancer functions was provided in Table 1. JAK/STAT signaling is known as the double-edged sword of cancer progression (Owen et al., 2019). Numerous studies summarized in this review illustrate that inhibition of JAK/STAT signaling by approved JAK inhibitors has potential anti-tumor effects, although those drugs were primarily designed for against AIDs. While the JAK inhibitors approved for AIDs treatment have not yet received approval for cancer therapy. Certain drugs, such as Ruxolitinib, have undergone clinical trials to assess their anti-tumor efficacy against various solid tumors. These trials have included pancreatic, head and neck cancers, as well as hematological tumors, and have shown positive responses. Pacritinib has also been investigated in clinical trials for its anti-tumor effectiveness against AML and CRC. Additionally, combining these JAK inhibitors with other drugs to achieve synergistic anti-tumor efficacy also been explored in clinical trials. More and more JAK inhibitors have undergone extensive research to assess their potential efficacy against tumors in animal models and clinical trials. These findings are paving the way for their future clinical applications in cancer treatment.

**TABLE 1:** Summary of JAK inhibitor drug for AIDs explored in cancer therapy.

Name	Targets	Approved for anti-AIDs	Explored anti-cancer efficacy
Ruxolitinib	JAK1, 2	MF, PV, GVHD	pancreatic cancer, head and neck cancer, breast cancer, lung cancer, hepatocellular cancer, T-ALL, CNL, aCML, MPN
Pacritinib	JAK2, JAK2V617F	MF	AML, GBM,CRC, lung cancer, prostate cancer, breast cancer, NPC
Fedratinib	JAK2, JAK2V617F	MF	PDAC, NSCLC, AEL, oral cancer cell
Tofacitinib	JAK1, 2, 3	RA, PsA, UC, JIA, AS	T-ALL
Baricitinib	JAK1, 2	RA	T-ALL, prostate cancer
Abrocitinib	JAK1, 2	AD	N.D. (no data)
Filgotinib	JAK1	RA	NSCLC
Oclacitinib	JAK1	CAD	pulmonary metastasis
Peficitinib	Pan-JAK	RA	epithelial ovarian cancer
Upadacitinib	JAK1	RA, PsA, AD, UC	NSCLC, lung cancer cells, breast cancer
Deucravacitinib	TYK2	PP	MPNST
Delgocitinib	JAK1, 2, 3, TYK2	AD	N.D.
